# Education and Culture as Correlated to the Health and Diseases of Women

**Published:** 1890-02

**Authors:** 


### Book Reviews.

---

Education and Culture as Correlated to the Health and Diseases of Women. By A. J. C. Skene, M. D. Geo. S. Davis & Co., Detroit, Mich. 1889.

This little volume forms a part of "The Physician's Leisure Library," and is one of a number of valuable works which have appeared in this series. Like everything else which has come from Skene's pen, it is well worth the reading. It is a plea for more rational training of girls during the period of sexual development. The subject is considered in a reasonable, dispassionate manner, and is free from the tendency to run to fads and hobbies, which characterizes most writings which have the same end in view. The author has studied the subject from personal observations and deductions, and not in the light of preconceived theories and *a priori* reasoning. His views, therefore, are entitled to more consideration than those of the majority of writers who seek to reform the manners and habits of their fellow-creatures. There is nothing impracticable or visionary in what he teaches, but all that he says commends itself to the reason and to common sense. We take pleasure in commending the work, not only to the profession, but to the laity as well, since it is the non-professional portion of the community that will be specially benefited by its teachings.

The low price at which the book is sold, twenty-five cents, is a minor point in its favor. V. O. H.

